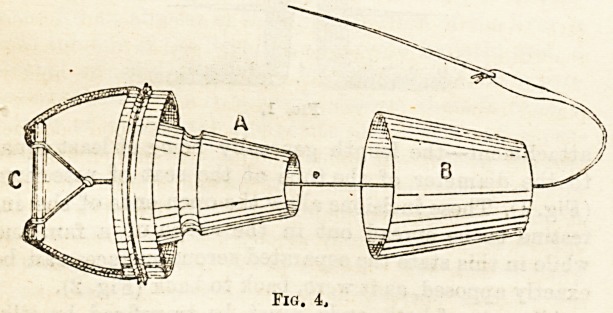# Progress in Surgery

**Published:** 1898-03-19

**Authors:** 


### PROGRESS IN SURGERY.

### SURGERY OF THE INTESTINES.

(*Continued from page* 416.)

**Multiple Constrictions of Tubercular Origin** generally occur, according to Hofmeister,[8] in the ileo-cæcal region. The hypertrophic form of inflammation almost always causes a narrowing of the lumen, while such a change is seldom observed as the result of the usual circular ulceration. The distance between the strictures varies from a few centimetres to one and a half metres. The length also varies—in two cases it was as much as eight centimetres. Where the contractions are localised in a relatively short segment of the intestine resection of the whole tract is desirable. Where two strictures are present some distance apart, with healthy intestine in the interval, the ideal method is to resect each stricture individually, but the patient is generally too debilitated to allow of this. Where the number of the strictures prevents their individual resection, and the extent of their distribution necessitates the sacrifice of a considerable length of intestine, entero-anastomosis yields the best results. Some cases, however, are too extensive to permit of this, and then nothing can be done, unless an attempt at relief be made in the case of some of the narrowest constrictions by treating them on the principles of the Heineke-Mikulicz pyloroplasty.

**Intestinal Anastomosis** by many new methods is reported. Cheatle[9] proposes the following: After removal of the affected segment of the intestines by transverse section, and its V-shaped piece of attached mesentery, the open ends of the gut lie requiring suture. Through all the coats forming the wall of each free end a longitudinal incision is made, and in each case it should be situated opposite the mesenteric

FIG. 1.

attachment—the length generally being at least equal to the diameter of the tube at the seat of resection. (Fig. 1). These incisions allow the open ends of the intestine to be spread out in the shape of a fan, and while in this state the separated serous surfaces can be exactly apposed, as it were, back to back (Fig. 2).

All coats of both ends must be transfixed by silk-bearing needles, and thus firmly joined together. All that then remains to be done is to suture the longitudinal incision that is left by Lembert or other suture.

Great care must be taken at the site of the four corners at the centre of the longitudinal incision (Fig. 3).

In using this method for ileo-cæcal junction there will be on each side of the joined intestines two equal-sized

Fig. 2.

superfluous flaps of large intestine. A portion of each of these flaps can be cut away in a V-shape, and the remaining part of these two edges can be inverted by the Lembert suture, so that the colon tapers to its union with the ilium. An almost identical method of operating to the above is given by Hartigan,[10] which he had formerly described in 1892. Several new bone bobbins are reported. Maunsell[11] describes one which consists of A (Fig. 4), a "button," with conical hollow stem and wide undercut flange; B (Fig. 4), a hollow cone, made to fit loosely over stem of A, and of such

Fig. 3.

length that, as it sits in the undercut of the flange, its apex projects slightly beyond the apex of A; and C (Fig. 4), two short bands of ordinary thread-covered elastic, joined in middle by short, thin ivory bar. The free ends of elastic are tied on either side into the groove on the flange of A. A silk thread is fastened to the bar after the manner of a trapeze, and then passed through both cones and threaded on a needle. A and B are decalcified, but the bar C is not. To introduce, first run a purse-string suture loosely around either cut

Fig. 4.

end of the intestine. Then pass the needle of C from within out about two and a half inches from the cut end of the lower intestine. Next let the cone B slip into the same intestine and leave it for some time. Tie the lower intestine by means of a purse-string suture into the groove on the stem of A, and then tie the upper intestine in the same way. Now steady A through the intestinal wall, and shove up B until it inverts both intestines into the flange of A. When satisfied that apposition is good push the ivory bar of C into lumen of A, and pull the silk thread until the bar is pulled out at the apex. Now, on account of the trapeze, and the even counterpull of the elastic bands, the bar rights itself, and, when the thread is slackened, rests securely across the apex of B, thus pushing the base of B firmly into the flange of A. Then the thread should be pulled short, and cut off. Gil[12] recommends the somewhat similar use of pieces of decalcified ivory with central apertures, turned in concavo-convex shapes and of different sizes, from eight millimetres to six centimetres in diameter—27 sizes in all. One piece is furnished with a short hollow stem or shank, to which the other piece is adjusted. The thickness of the pieces is proportioned to their circumference. Besides the central hole, there are two other very small orifices, through which thin elastic bands pass that serve to join the pieces, thus forming a disc when they are approximated. These bands exercise a moderate pressure intended to produce atrophy of the tissues placed between the two pieces, while not causing gangrene of the intestines. Ball[13] quotes four cases in which he has used a decalcified bone or ivory ring made in three sizes, which are sufficient for all ordinary cases. The centre of each is perforated to allow of the passage of intestinal contents, the upper and lower ends are smoothly rounded off, while round the circumference a deep groove is turned, wide enough to allow involution of the cut edges of the intestine, while keeping the peritoneal surface in tolerably close contact; a considerable undercutting of this groove provides accommodation for any surplus of involuted intestine. The chief feature in the application of this ring is, however, the fact that a continuous lacing suture, connecting both portions of intestine, is passed loosely through the entire circumference of the divided ends of bowel before the ring is placed in position. When the circuit is complete, a couple of the loops are gently separated, and the ring gently slipped into position. By gently pulling the suture loop by loop, as you would the lace of a boot, it is gradually tightened into the groove of the ring, at the same time drawing in and involuting the cut edges of the intestine, and bringing their peritoneal surfaces into contact. When all is nicely adjusted the two ends of the suture are firmly tied and a second fine continuous suture is passed round the outside through the peritoneum alone, when the two surfaces of this membrane are in apposition. Pilcher[14] follows Ullmann by adopting Maunsell's method, but inserted a spool of raw potato into the approximated intestines, brought out through a longitudinal lateral incision, and united the two ends by a single encircling thread of catgut or silk. The bowel is then disinvaginated in the ordinary way and the lateral incision closed.

**Perforating Typhoid Ulcer** has been treated, according to Finney,[15] by laparotomy in 47 cases with 13 recoveries. The best time for operating is as soon as the patient has recovered from the shock attending the perforation—usually in a few hours. It occurs in from 1 to 2 per cent. of all cases, and as a cause of death in fatal cases it is found in between 6 and 7 per cent. It occurs in the ileum in over 80 per cent. of all cases, in the large intestine in 12 per cent., and the vermiform appendix in 5 per cent. In eight of the cases the diagnosis was wrong. Finney places most reliance on severe continued abdominal pain with nausea and vomiting, and on a marked increase of leucocytes. During typhoid fever there is no leucocytosis, but it occurs immediately after perforation. Thus in three cases the numbers before and after were 8,300 and 24,000, 6,500 and 10,600, 3,000 and 16,400. Monod and Vanverts[16] consider the prospects of operative treatment as unfavourable when the perforation has occurred at a late stage of typhoid fever, and particularly during convalescence, or at the end of a relapse. Surgical intervention would in most cases consist in median laparotomy and simple suture of the perforation. Intestinal resection and the formation of an artificial anus would be reserved for cases in which the lesions are more or less complicated. Panton[17] gives a successful, and Davies[18] an unsuccessful, case.

**Intussusception.**—Gibson[19] has tabulated 239 acute cases. The total mortality is given as 53 per cent., and is essentially due to the duration and cause of the obstruction. The longer the case is left before operation the greater the irreducibility, and the higher the mortality. The writer is far from believing that mechanical distension should be altogether discarded, but would very much limit its application. Many instances have occurred where a deceptive success has followed inflation, before resort was finally had to abdominal section. Cases of successful operation for chronic intussusception are reported by Barrow,[20] and Wright and Knowles.[21] The latter showed an extremely rare condition of an intussusception of the appendix vermiformis into the cæcum.

[8] Annals of Surg., Sept., 1897, p. 393. [9] Lancet, May 22, 1897, p. 1,403. [10] New York Med. Journ., July 17, 1897, p. 69. [11] Brit. Med. Journ., May 29, 1897. [12] Lancet, Aug. 28, 1897. [13] Brit. Med. Journ., Aug. 24, 1897, p. 1,021. [14] Annals of Surg., Oct., 1897, p. 485. [15] Therapeut. Gazette, May 15, 1897, and Lancet, July 10, 1897. [16] Brit. Med. Journ., June 12, 1897. [17] Annals of Surg., Aug., 1897, p. 219. [18] South African Med. Journ., Aug., 1897, p. 94. [19] New York Med. Rec., July 17, 1897, p. 73. [20] Lancet, May 22, 1897, p. 1,411. [21] Brit. Med. Journ., June 12, 1897, p. 1,470.

### RECTUM AND ANUS.

**Fistula in Ano.**—In an article upon the treatment of fistula Mr. J. E. Platt lays stress on several points already recognised to some extent. It may be taken as a general rule that all secondary sinuses and diverticula should be laid open. If the sinus extend above the internal opening it should be laid open if submucous. After scraping, a "back-cut" is often useful. Tight packing is unnecessary and harmful. The average time given for recovery is seven weeks in non-tuberculous and nine weeks in tuberculous cases. With regard to the treatment by excision of the sinus with immediate suture experience seems to be somewhat disappointing, and opinions widely differ. Free division of the anterior part of the sphincter ani in women is apt to lead to permanent incontinence, owing to its partial decussation with the sphincter vaginæ. Double, too deep, or oblique incision of the sphincters is also apt to cause incontinence.[1]

**The Rectum as a Reservoir.**—Arguments in favour of regarding the rectum as a temporary receptacle for fæces are given by Dr. Bodenhainer, from its form and structure. The rectum is not a pouch in infancy, but a plain tube; it only becomes a pouch when there is voluntary control over the sphincter. The writer differs from O'Beirne, who stated that the rectum is always empty; for he finds it the rule that there are commonly some fæces in the rectum, and this is the experience of gynæcologists. He questions the value of the superior sigmoid sphincter of O'Beirne and the rectal sphincter tertius of Hyrtl.[2]

**Prolapse, &c., of the Rectum.**—The treatment of this condition is the subject of a paper by S. Fowler, of Brooklyn.[3] Tonics, cold-water enemata, and defæcation in the horizontal position are recommended as palliative measures. Children should have the buttocks strapped together with plaster. For operative treatment, linear cauterisation with the cautery or fuming nitric acid is recommended in slighter cases; but for severe cases one of the following operations may be required: (1) Subcutaneous purse-string suture around anus; (2) excision of parts of rectal wall; (3) Gersung's torsion method (after excision); (4) fixation; (5) amputation.

For the treatment of acute prolapsus ani, Dr. Frank Elvy recommends the use of a full-sized Tait's cervical dilator as a pessary to keep the bowel back, keeping it in position by a collar of dentist's wax, supported by cotton wool, and a firmly-applied T-bandage. This treatment has been very successful in his hands.

With regard to the extensive operation proposed by Jaennel in 1889 for colopexy, it is, perhaps, doubtful whether it can be regarded as legitimate surgery unless the patient's condition is such as to warrant the risks both immediate and remote. A paper on the subject was read by Dr. Joseph Bryant (New York).[4] The intention of the original operation was to afford rest to the rectum by instituting an artificial anus; this has been extended to the fixation of the gut to the abdominal wall. The method suggested is to make an abdominal section parallel to and above Poupart's ligament, and through the peritoneum, which is separated for some little distance round the wound. The gut is then drawn well up into the wound, and the peritoneum sewn to it. Then stitches are inserted through the parietes, also including the bowel, and are tied. The abdominal scar is apt to be weak afterwards. Of 29 cases there was non-recurrence within comparatively short periods in 22, and there was no mortality. In the discussion which followed opinions were generally in favour of local treatment of the rectum by preference.

**Cancer of the Rectum.**—The interest attached to this important subject is naturally unabated. Dr. Quénu has studied its pathological anatomy,[5] and shows that the part intervening between the anus and rectum may give rise to squamous or cylindrical-celled cancer according to circumstances. He gives four classes: (1) Anal cancer; (2) infraperitoneal cancer; (3) highly-placed cancer; (4) general rectal cancer. Whatever the exact site may be, the perirectal fat soon becomes affected by a dense thick œdema. In anal cancer the superficial inguinal glands are affected, and in rectal cancer the hæmorrhoidal glands. During the last 12 years Kraske has had an experience of 110 cases, 80 of which were subjected to operation.[6] The two sexes were nearly equally affected. The majority of the cancers were of the cylindrical celled variety and in the upper part of the rectum. Squamous anal cancers are the more malignant. The early stages are characterised by an entire absence of symptoms. With regard to treatment, it is of the first importance to define the limits of the position and of the infection of the growth as far as possible, particularly the latter. The curative results by operation are improving from year to year, and Kraske condemns routine colotomy. Adhesions low down do not contraindicate operation. Preparatory treatment of the patient is all-important. Kraske regards the osteoplastic operation on the sacrum as fraught with danger, inasmuch as the central sacral canal is opened up and free drainage is restricted. He has reduced his mortality from 40 per cent. to less than 10 per cent. Several cases of Kraske's operation are recorded by Dr. W. W. Keen (Philadelphia).[7] Dr. Bell (Montreal) estimates the freedom from recurrence within four years at 10 per cent. Perhaps these figures indicate the best results of the best surgeons. Mr. Cheyne collected 100 cases by eminent operators, and found that over 60 per cent. would have been better off if colotomy alone had been done, and 27 died as an immediate effect of operation. Mr. W. Rose fully agrees with this experience, and says from a careful consideration of all the facts one is driven to the conclusion that excision of the rectum must be laid aside as a routine procedure.[8] He advocates colotomy at a very early stage of the disease.

In the female the advantages of resection of the rectum through the vagina with restoration of continuity of the rectum are great. Dr. Heydenrich (Nancy) has collected and described such cases. The recto-vaginal septum may be divided right through, or flaps dissected off, but this latter makes the operation more difficult. The peritoneum is not opened. The vaginal operation is particularly applicable to cancers which are situated not very high up.[9]

[1] Medical Chronicle, Oct. 1897. [2] N. Y. Med. Gaz., Oct. 30 and Nov. 6, 1897. [3] Treatment, Aug. 12, 1897. [4] Lancet, Dec. 11, 1897. [5] Annals of Surgery, Aug. 1897. [6] Annals of Surgery, Dec., 1897. [7] Annals of Surgery, Sep., 1897. [8] Therapeutic Gazette, May 15, 1897. [9] Practitioner, July, 1897.

## Figures and Tables

**Fig. 1. f1:**
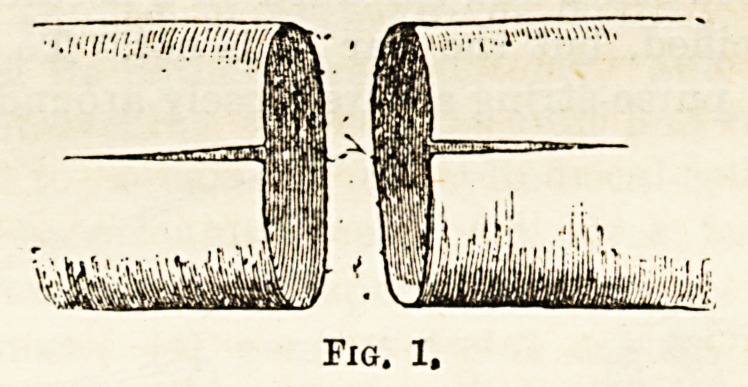


**Fig. 2. f2:**
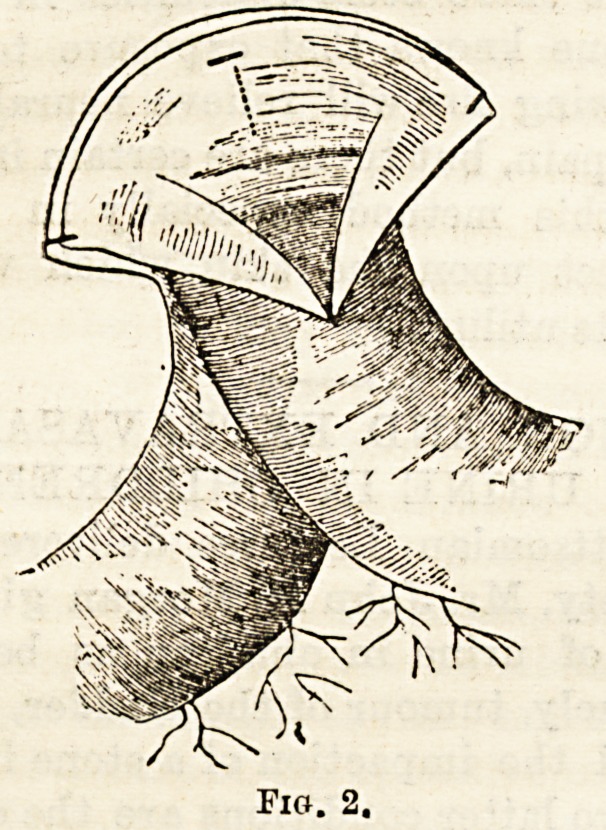


**Fig. 3. f3:**
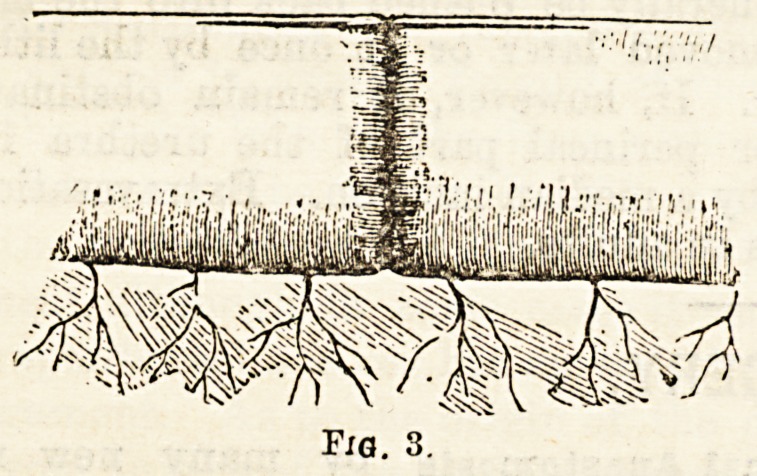


**Fig. 4. f4:**